# Fabrication of an efficient vanadium redox flow battery electrode using a free-standing carbon-loaded electrospun nanofibrous composite

**DOI:** 10.1038/s41598-020-67906-6

**Published:** 2020-07-07

**Authors:** Mahboubeh Maleki, Gumaa A. El-Nagar, Denis Bernsmeier, Jonathan Schneider, Christina Roth

**Affiliations:** 1 Institute for Chemistry and Biochemistry, Freie Universität Berlin, 14195 Berlin, Germany; 2 Chemistry Department, Faculty of Science, Cairo University, Cairo, 12613 Egypt; 3 Institute of Chemistry, Technische Universität Berlin, 10623 Berlin, Germany; 4 Electrochemical Process Engineering, Faculty of Engineering, University of Bayreuth, 95447 Bayreuth, Germany

**Keywords:** Energy, Batteries

## Abstract

Vanadium redox flow batteries (VRFBs) are considered as promising electrochemical energy storage systems due to their efficiency, flexibility and scalability to meet our needs in renewable energy applications. Unfortunately, the low electrochemical performance of the available carbon-based electrodes hinders their commercial viability. Herein, novel free-standing electrospun nanofibrous carbon-loaded composites with textile-like characteristics have been constructed and employed as efficient electrodes for VRFBs. In this work, polyacrylonitrile-based electrospun nanofibers loaded with different types of carbon black (CB) were electrospun providing a robust free-standing network. Incorporation of CBs (14% and 50% weight ratio) resulted in fibers with rough surface and increased mean diameter. It provided higher BET surface area of 83.8 m^2^ g^−1^ for *as-spun* and 356.7 m^2^ g^−1^ for carbonized fibers compared to the commercial carbon felt (0.6 m^2^ g^−1^). These loaded CB-fibers also had better thermal stability and showed higher electrochemical activity for VRFBs than a commercial felt electrode.

## Introduction

The need for modern and effective grid-connected energy storage systems is growing worldwide due to the expansion of only intermittently available renewable energy sources and the inherent request for services of power quality and energy management. Redox flow batteries (RFBs), especially all-vanadium RFBs (VRFBs), have been considered as promising stationary electrochemical storage systems to compensate and stabilize the power grid. This is attributed to their unique features such as their flexibility, scalability, independently scaled power and energy, high efficiency, room temperature operation, and extremely long charge/discharge cycle life^1–3^.

The electrodes are the essential components of the VRFBs since their morphological, structural, chemical and physical properties affect the overall improved performance, durability, efficiency and even the total cost of the VRFBs technology^4^. Currently, the most commonly used materials for electrodes are carbon-based materials including carbon cloth, carbon-polymer composite, graphite felt, carbon paper and graphene^5^ thanks to their low cost, safety, stability and their inertness. However, their relatively low electrochemical activity towards the vanadium redox reactions together with their poor wettability limit the battery power density.

In this perspective, several strategies have been introduced to overcome the mentioned issues and to improve the carbon-based materials performance. These strategies are mainly based on acid, thermal and (electro-)chemical treatments as well as surface modifications by functionalizing the surface with hetero-atom functional groups (i.e. OH, SH, NH_2_) or by surface coating with catalysts such as Bi, Mn, Ir or metal oxides (PbO_2_, CeO_2_, Mn_3_O_4_, ZrO_2_, Ce_0.8_Zr_0.2_O_2_, Nd_2_O_3_, etc.)^6–13^. For instance, surface modification by boiling in concentrated sulphuric acid for 5 h led to a dramatic improvement in the electrocatalytic activity of the graphite felt electrode due to the formation of oxygen functional groups and thus increased hydrophilicity of the felt surface that enhanced the effective surface area^14^. Likewise, the electrochemical activation of the graphite felt at oxidation degrees of 560 and 840 mA h g^−1^ improved the formation of the oxygen-containing surface groups that led to a significant improvement in Coulombic efficiency, voltage efficiency, and energy efficiency of the cell^15^. Wang et al.^16^ reported that nitrogen-doped carbon nanotubes (N-CNT) grown on the carbon felt significantly enhanced the battery performance. They attributed this to the alteration of the chemisorption characteristics of the vanadium ions that could generate defect sites. These sites were electrochemically more active and could increase the oxygen species and thus the N-CNT was electrochemically more accessible than the CNT. Wu et al.^17^ reported that Nitrogen-doped carbon nanospheres grown on graphite felt exhibited excellent electrocatalytic activity and electrolyte wettability for the vanadium ion redox reaction. As a result, superior battery performance in terms of energy efficiency and capacity retention was achieved.

Additionally, Fetyan et al.^18^ found that modification of the commercial carbon felt (CF) with Nd_2_O_3_ had a catalytic effect towards both redox couples, V^4+/^V^5+^ at the positive and V^2+^/V^3+^ at the negative side, and these modified felts maintained their oxygen-donating functionalities on the surface after exchanging the electrolyte after 50 cycles. Moreover, the thermal treatment of the commercial carbon felts at 400 °C in air for 30 h resulted in significant improvements in performance of the vanadium cell^19–21^. In these investigations, increased activity of the electrode is generally attributed to enhanced oxygen content on the surface^20,22,23^.

On the other hand, another efficient alternative approach is to replace the conventional carbon felt electrodes by an electrospun modified nanofibrous network. This 1D morphology of nanofibers within a 3D porous network has exceptional properties such as high tensile strength, high specific surface area, high porosity, and unique electrical, mechanical and physical properties leading to better performance in flow batteries^5,24^.

The electrospinning (ES) technique is one of the most efficient procedures to prepare these free-standing and robust nanofibrous mats with controlled morphological properties which then can be used in various applications^25–29^. The high aspect ratio, reproducibility and simplicity make this technique a very attractive fabrication strategy for tailoring self-standing nanofibrous mats^30^. The electrospun nanofibers possess unique characteristics such as high surface-to-volume ratios, controllable fiber diameters and various surface morphologies (i.e. solid, hollow, core–shell and porous)^31–33^.

Polyacrylonitrile (PAN) is the most popular host polymer precursor for producing free-standing electrospun carbon nanofibrous electrodes (CNFs) and has been used alone^34,35^ or blended with other polymers (e.g. polyvinylpyrrolidone^36^) to fabricate electrospun fibrous electrodes for VRFBs. In our previous work^34^, PAN-based CNFs were successfully produced and used as free-standing electrodes for VRFBs. However, using pure PAN precursor for the creation of the carbon nanofibers may be considered quite expensive for practical upscaling without providing adequate electrochemical performance. Moreover, many researchers have started embedding PAN with other components (e.g. carbon black^37^, multi-wall carbon nanotubes^38^, TiO_2_^39^, CeO_2_^40^, ZrO_2_^41^) to improve the electrochemical behaviour in a composite form. For example, Wei et al.^38^ reported the enhanced electrochemical activity of the loaded PAN-based CNFs with carbon nanotubes compared to the pristine CNFs but unfortunately, the poor loading capacity of PAN hinders higher carbon uptake. Furthermore, various pre-treatment strategies (i.e. acid, electrochemical and thermal treatment) resulted in an altered performance of the carbon felt towards VRFBs^42^.

On the other hand, carbon black (CB) filled polymers are widely used in industrial applications owing to their cost advantage over other fillers to improve the mechanical, electrical, or thermal properties of the materials^43–45^. In our recent publication, polyacrylic acid (PAA) was used as a binder for higher CB loading within PAN-based CNFs and the obtained CNFs exhibited promising results as electrodes for VRFBs^37^.

However, there is no deep understanding from a material point of view when CB is added to the polymeric matrix utilized in the form of nanofibrous mat as electrode of VRFBs. The key properties of carbon blacks are considered fineness (primary particle size distribution), structure (aggregate size/shape), electrical conductivity, porosity and surface chemistry which all depend on the manufacturing process and may have an influence on the CB loading degree within fibers as well as their performance in VRFBs.

Here, in-situ electrospinning is used to fabricate nanocomposites constructed of PAN-based nanofibers loaded with different types of commercially available carbon black powders. Moreover, the influence of thermal post-treatment (mild and severe heat treatment at 300 °C and 1,000 °C, respectively) on the produced carbon black-loaded nanofibrous electrodes is investigated using various characterization techniques such as SEM, BET evaluation, Raman spectroscopy, TGA, cyclic voltammetry and full-cell cycling test. The main goal of this study was to design and fabricate novel nanofibrous electrodes with tailored properties for VRFBs application with simultaneously correlating the physical and structural properties of the electrospun electrodes to their obtained electrocatalytic activity.

This study establishes guidelines for a successful fabrication of free-standing carbon black-loaded 3D nanofibrous mats as electrodes with good electrochemical behaviour in VRFBs using electrospinning. The results will help to understand the role of the carbon black type and its loading within the electrospun nanofibers as well as mild/severe heat treatment on the electrochemical behaviour of an efficient electrode for VRFBs. In the future, an upscaled approach for the fabrication of carbon-filled electrospun materials seems feasible.

## Materials and methods

### Materials

Polyacrylonitrile (PAN; Mw 150,000), Poly (acrylic acid) (PAA; Mv 450,000) were purchased from Sigma-Aldrich, Germany. Carbon Black CB-L-1 (XC-max) and CB-H-1 (XC-72) were obtained from Cabot Corporation, Latvia. CB-L-2 (Ketjenblack EC600JD) was obtained free of charge from Fraunhofer UMSICHT, Germany and CB-H-2 (Printex L6 powder) was received from Orion Engineered Carbons GmbH, Germany*.* N,N‐dimethylformamide (DMF) was purchased from Carl Roth, Germany. Nafion Dispersion (alcohol based at 20% weight) was received from Ion Power, USA. When needed, milli-Q water with ~ 18.2 MΩ cm^−1^ was used. Anion exchange membrane (FAP 450) was obtained from Fumatech, Bietigheim-Bissingen, Germany. Commercial vanadium electrolyte (1.6 M total Vanadium, 2 M H_2_SO_4_, 0.015 M H_3_PO_4_, Batch-No.: 207445) was recieved from GfE Gesellschaft für Elektrometallurgie mbH, Nürnberg, Germany. All the used chemicals were ultrapure and were used as received without any further purifications.

### Fabrication of nanofibrous composite by electrospinning

#### Electrospinning solution preparation

The spinning solvent was N,N-dimethylformamide (DMF) and solutions were prepared by stirring polymers in the solvent. First, the polymers (i.e. blend of PAN and PAA) were dissolved overnight in DMF and then, CB was added to the obtained polymeric solution and stirred for another day. Different carbon blacks (detailed in Table S1) with various properties were used: CB-L-1, CB-L-2, CB-H-1, CB-H-2. All sample preparations were carried out under ambient conditions. ES was done using a commercially available unit (EC-DIG, IME Technology).

#### Fiber fabrication using electrospinning

The ES solution was placed in a 2 mL syringe (B-Braun, Germany) with a 21-gauge needle mounted on a syringe pump. The syringe pump was used to provide a constant stream of solution at the tip of the needle. Electrical potentials of + 15 kV and − 2 kV were applied to the needle and collector by a positive and a negative power supply, respectively. The fibers were collected at a distance of ~ 14 cm from the needle on a grounded copper disk with a diameter of 40 mm. Spinning was done under ambient conditions. Later on, the obtained fibrous mats were vacuum dried for 1 day to ensure the removal of all solvents and then stored at room temperature before conducting further tests.

#### Thermal treatment process

Thermal heating was used as a post-treatment after in-situ electrospinning to further enhance the electrochemical properties of the created fibrous electrodes. Two strategies of thermal treatment were pursued to modify the electrospun mats for VRFBs: (i) a mild heat treatment at 300 °C to produce a flexible polymeric matrix with loaded CBs; and (ii) a severe heat treatment by carbonization at high temperature (1,000 °C) to make a rigid carbon- nanofibrous matrix.

In the first strategy, the nanofibrous mat was placed in an oven (Linn High Therm, Germany) held at a constant temperature of 300 °C for 24 h. In the second strategy: the collected electrospun fibers were first stabilized by heating them at 280 °C with a heating ramp of 1 °C min^−1^. The temperature was held for 1 h in an open tube in a conventional furnace (split tube furnace EST 12/300, Carbolite Ltd). The stabilized fibers were then carbonized under a nitrogen flow by heating them to 1,000 °C at a rate of 5 °C min^−1^ and the temperature was held for 1 h.

### Characterization of electrospun nanofibrous composite

Fiber and mat morphologies were analyzed with the aid of field emission scanning electron microscopy (SEM; Hitachi UHR FE-SEM SU8030) after gold sputter coating (Safematic CCU-010 HV) of the samples for 30 s (~ 5 nm). The average and standard deviation of the fiber diameters were measured by choosing 100 fibers images and analyzing them using the IMAGEJ software (v.1.52a, National Institute of Health, USA). Scanning transmission electron microscopy (STEM-in-SEM) was conducted at an operating acceleration voltage of 30 kV. STEM samples were prepared by directly depositing the *as-spun* fibers on a carbon-coated copper grid.

The specific surface areas of the obtained fibers were determined by a BET evaluation of Krypton physisorption measurements using a Quantachrome Autosorb-iQ instrument. Prior to the physisorption analysis, the fibers were degassed at 85 °C for 24 h under vacuum in order to remove the adsorbed impurities and moisture. Pore size distributions were derived from N_2_ physisorption measurements evaluated with a QSDFT kernel (adsorption branch kernel at 77 K based on a slit-pore model (pore diameter < 2 nm) and a cylindrical pore model (pore diameter 2–5 nm) and a spherical pore model (pore diameter > 5 nm).

The electrochemical properties of the electrode were investigated using cyclic voltammetry (CV) in a three-electrode cell setup by using a Galvanostat/Potentiostat Reference 600 (Gamry Instruments) at room temperature. The working electrode consisted of a sample with the dimensions of 5 mm × 5 mm sandwiched between two glassy carbon plates (50 mm × 10 mm × 1 mm, Sigradur, HTW). A commercial carbon felt (CF, 100 mm × 10 mm × 6 mm, SGL GFA6 EA) was used as the counter electrode and a saturated calomel electrode (SCE) was used as the reference electrode. The weight of all samples utilized as working electrode was ~ 8 mg. The cell was filled with 0.2 mol L^−1^ vanadium (51% V^3^^+^ and 49% V^4^^+^) in 2 mol L^−1^ H_2_SO_4_ electrolyte. The CV measurements were performed over a potential range from 0.2 to 1.2 V at a scan rate of 2 mV s^−1^.

Raman spectroscopy was performed with a REN-ISHAW via Raman spectrometer with a Leica microscope using 633 nm laser as an excitation source, 60× optical lens and stream-line mode to gain more detailed information about the surface defects of the as-prepared electrospun nanofibers. Deconvolution of the spectra was performed by mixed Gaussian/Lorentzian peaks to describe both the main D- and G-bands and the three minor ones D2, D3 and D4 using Origin Pro 2017.

The thermal stability of the fabricated mats was investigated with thermogravimetric analysis (TGA; Netzsch STA 449F5). The runs were performed in the presence of inert gas, at a heating rate of 10 °C min^−1^ and at temperatures ranging from room temperature to 1,000 °C.

Full-cell charge/discharge cycling was performed in a battery test system (857 Redox Flow Cell Test System; Scribner Associates, Southern Pines, NC, USA). A custom made 4 cm^2^ flow through cell, equipped with an anion exchange membrane (FAP 450) and graphite plates as current collectors, was used. On the positive side, two sheets of commercial felt (GFD 2.5, SGL Carbon, Germany) were sandwiched together and served as electrode. On the negative side, either the carbonized electrospun material (loaded with CB-L-2 and sandwiched with one sheet of commercial CF to make an overall electrode thickness of 5 mm) or two sheets of commercial CF (as reference material) were used. Upon installation, the electrodes were compressed by 20%. 50 mL of a commercial vanadium electrolyte (1.6 M total Vanadium, 2 M H_2_SO_4_, 0.015 M H_3_PO_4_) per side were pumped at a flow-rate of 10 mL min^−1^. The system was kept under a constant flow of Argon gas to ensure inert conditions. Galvanostatic cycling was performed within the voltage limits of 1.65 V and 0.8 V for charging and discharging, respectively with applied current densities of 25 and 50 mA cm^−2^ and five cycles at each current density.

## Results and discussions

### Fiber fabrication

CBs with different morphological and physical properties were in-situ incorporated into the electrospun webs, as described in the experimental section. Two groups of carbon blacks with different functionalities (see Table S1) were used to fabricate CB-loaded nanofibrous composite: group L; low CB content, including L-1 and L-2 and group H; high CB content, including H-1 and H-2.

The optimized nominal weight ratio of CB to the polymers was ~ 14% for group L and ~ 50% for group H providing high CB content as well as electrospinnability. It is worth to mention that production of the electrospun nanofibers from solutions containing higher loading of CB beyond mentioned values was impossible because of the high solution viscosity and discontinuity of the flow when spinning. Therefore, CB-loaded nanofibers of group L were fabricated from ES solution of polyacrylonitrile (PAN), polyacrylic acid (PAA; used as binder for higher CB loading) and carbon black (CB) mixture with PAN:PAA:CB weight ratio of 77%:9%:14%, while group H nanofibers were created from mixture with 45%:5%:50% weight ratio (see Table S2 for the solution ratio).

**Figure 1:**
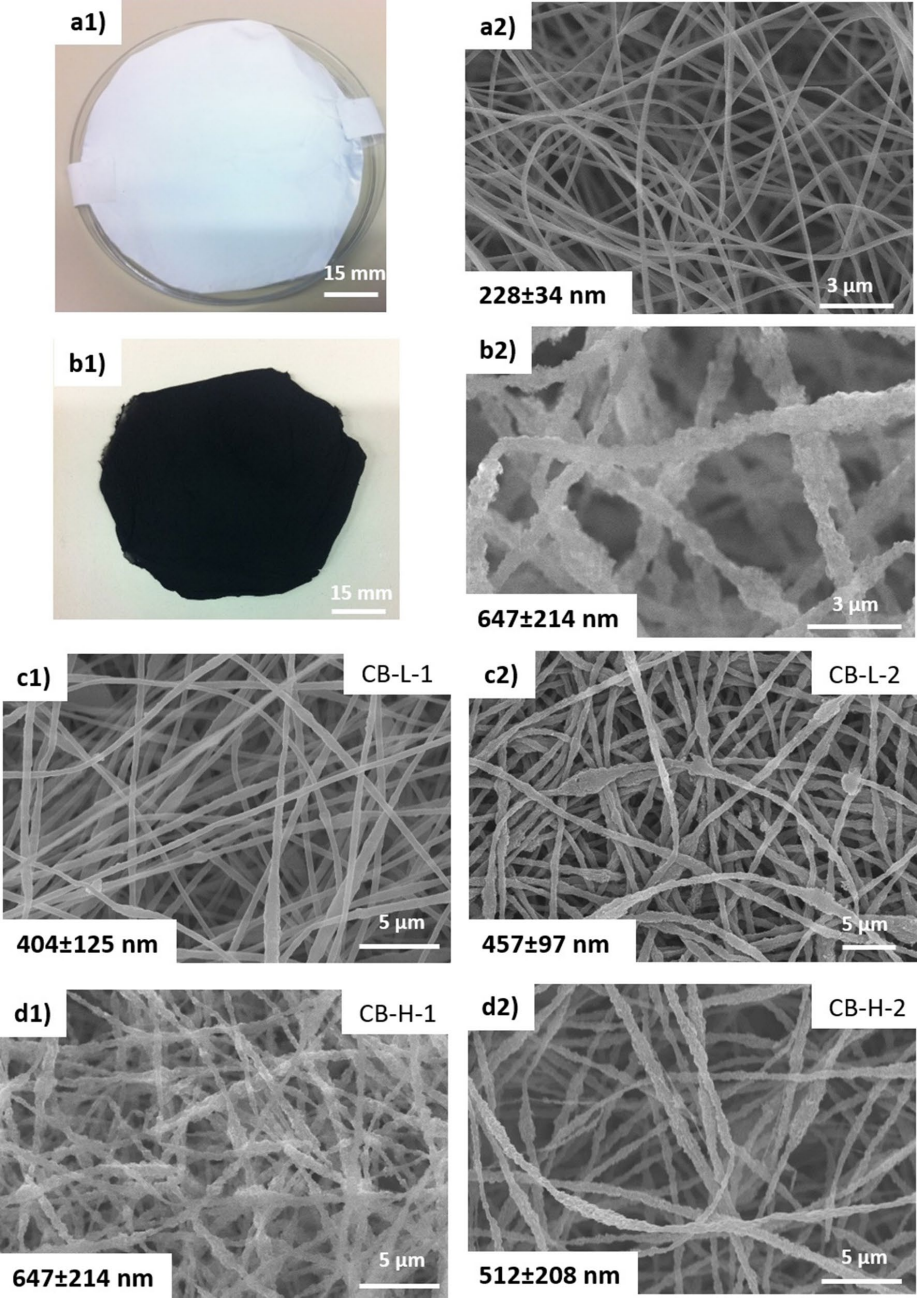
Optical and SEM images of *as-spun* nanofibrous electrodes made of (**a**) PAN (CB-free), (**b**) PAN loaded with CB-H-1 with weight ratios of 45% (PAN), 5% (PAA) and 50% (CB) respectively. SEM images of *as-spun* CB-loaded nanofibrous electrodes with CB contents of (**c**) 14% (group L) and d) 50% (group H).

Figure 1 displays selected optical and SEM images of the obtained *as-spun* pristine PAN-based and CB-loaded PAN-based nanofibers. The addition of CB to the fibers could be visually assessed from the change in the colour of the *as-spun* pristine fibrous mats from white (CB-free) to black (with CB) as obviously seen in Fig. 1a1, b1. These nanofibrous mats formed a randomly oriented 3D fibrous structure, as clearly shown in Fig. 1b2–d2. As seen in the optical images, both the pristine and CB-loaded fibrous sheets presented a smooth surface with continuous monolithic structure. However, when CB was loaded into fibers, the fiber surfaces became increasingly irregular and the agglomeration of the CB particles was visible on the fibers surface that could increase the surface roughness of the fibers.

During electrospinning, the CB particles were oriented along the driving voltage creating ‘streamlines’ in the PAN/PAA solution attributed to the elongation effect of the fluid jet. Consequently, they became parallel to the long-axis of the individual fibers^46^, resulting in small changes of the smooth cylindrical shape of the fibers (Fig. 1a2 vs. 1b2). Indeed, the average diameter of the obtained fibers significantly enlarged with the CB loading, where the fiber diameter increased from 228 nm for pristine fibers to 404 nm and 647 nm for the loaded CB-fibers with 14% and 50% CB, respectively. In fact, CB particles were thought to absorb and slow down the evaporation of the ES solvents as reported by Hwang et al.^47^, thus, an increased loading of CB up to 50% (CB-H) resulted in an increase in the fiber diameter.

**Figure 2:**
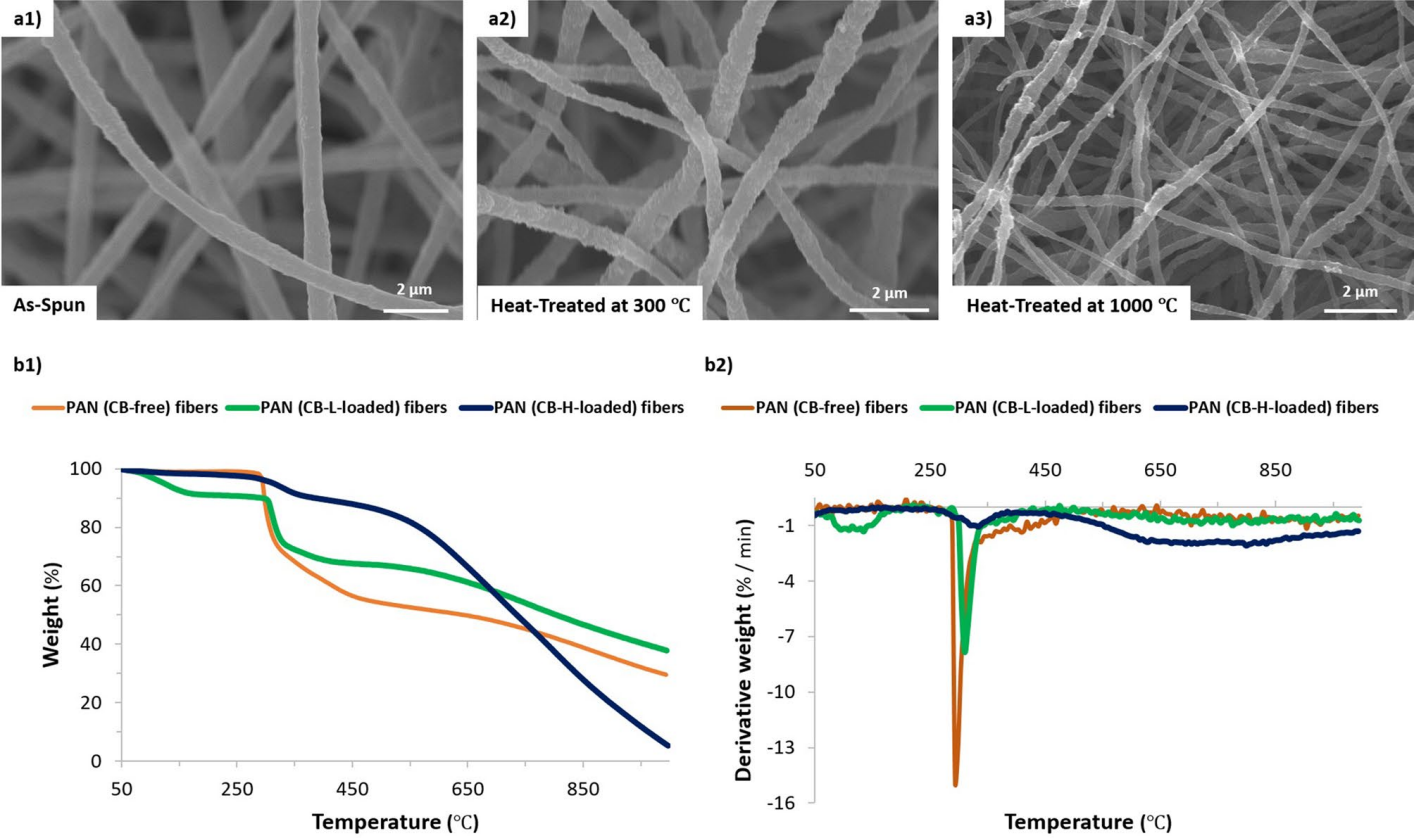
SEM images of PAN/PAA/CB-L nanofibrous electrode for (**a1**) *as-spun*, (**a2**) heat-treated at 300 °C and (**a3**) carbonized samples. (**b1**) TGA and (**b2**) derivative TGA thermograms of *as-spun* nanofibrous electrodes without CB and with CB: PAN (CB-free), PAN (CB-L-loaded) and PAN (CB-H-loaded) fibers.

Collected fibers were further heat treated to enhance their physical and electrochemical properties at two different temperatures 300 °C and 1,000 °C, as described in detail in the experimental section. The heat treatment using both strategies preserved the morphology and structure of the fabricated free-standing fibrous mat, as shown in Fig. 2a. However, the heat treated fibrous mat at relatively low temperature (at 300 °C for 24 h) showed very flexible fibrous structure: it could be folded like a non-woven cloth and rolled up easily into a scroll. Unlike that the fibrous mat carbonized at high temperature (at 1,000 °C for 1 h) exhibited rigid behaviour without flexibility.

### Thermal stability

To investigate the effect of CB loading on the thermal stability of the produced nanofibrous composite, TGA was performed on the CB-free samples (as reference) and the fibrous composite loaded with CB-L and CB-H. The changes in the weights of various electrospun fibers as a function of temperature are plotted in Fig. 2b.

The decomposition temperature was noted at 295 °C for the electrospun polymeric nanofibers made of PAN without any loaded CB. Electrospun pure PAN nanofibers exhibited a relatively large exothermic peak at about 295 °C, which originated from the complex and multiple chemical reactions (i.e., dehydrogenation, instantaneous cyclization and crosslinking) of PAN during the process of thermal treatment via the free radical mechanism that was in agreement with other findings^48,49^.

Surprisingly, the presence of CB within nanofibers shifted the exothermic peak to a higher temperature instantaneously with broadening it compared to that of the pure PAN fibers. That is, the loading of CB within PAN-nanofibers resulted in improving their thermal stability, as revealed from the increase of initial decomposition temperature from 295 °C for pristine PAN fibers to 310 °C for CB-L and 325 °C for CB-H loading into PAN nanofibers. The temperature for 40% weight loss of the CB-loaded nanofibers was ~ 670 °C for CB-L and ~ 685 °C for CB-H, which was ~ 255–270 °C higher than that of the pristine PAN nanofibers (i.e. ~ 415 °C).

As observed in the thermal analysis curve (Fig. 2b2), all the samples exhibited three main stages in the loss of their mass: the first (270–340 °C), the second (340–460 °C) and the third (600–750 °C), could be assigned to the thermal decomposition of used polymers. Moreover, a weak endothermic peak at ~ 70–120 °C corresponding to the loss of moisture appeared.

The broadening of the exothermic peak in the presence of CB, especially when a high content of CB-H was loaded into PAN-fibers, suggested that CB might modify the activity of the free radicals involved in the above-mentioned complex chemical reactions. This effect was previously reported for the addition of carbon nanofillers such as CB in the polymeric composites^50^. The decreased peak intensity could be due to the interactions between PAN and CB. Most likely, effectively dispersed CB could built up a particle network within the CB-polymer matrix, providing a physical interruption against the release of pyrolysis gases in the early stage of decomposition especially when CB-H with high loading of 50% weight ratio was used.

Fig. S1 shows TGA thermograms of the as-received different CB powders of both groups (CB-L and CB-H). As clearly seen in the Fig. S1, CB of group H exhibited a higher thermal stability under investigated temperature range, where no decomposition peaks were observed. On the other hand, there was a loss in mass covering the temperature range with the onset temperature of about 500 °C and 700 °C for CB-L-1 and CB-L-2, respectively. This mass loss most likely corresponded to the thermal degradation of some of their surface functionalities that were present in CB and this effect was unexpectedly stronger in CB-L compared to the CB-H.

### BET surface area

The BET surface areas of our electrospun nanofibrous mats were determined by physisorption measurements and were compared to the surface area of a commercial carbon felt. The specific surface area of the commercial carbon felt (GFA 3EA) was 0.6 m^2^ g^−1^. In comparison, *as-spun* electrospun nanofibrous mats of PAN (CB-free sample) and PAN/PAA/CB-H (CB-loaded PAN nanofibers) had BET surface areas of 15.5 and 83.8 m^2^ g^−1^, respectively. Indeed, the CB-loaded PAN nanofibrous mat exhibited ~ 140 and ~ 5.4 times higher specific surface area than the commercial carbon felt and pure PAN fibrous network, respectively.

**Figure 3:**
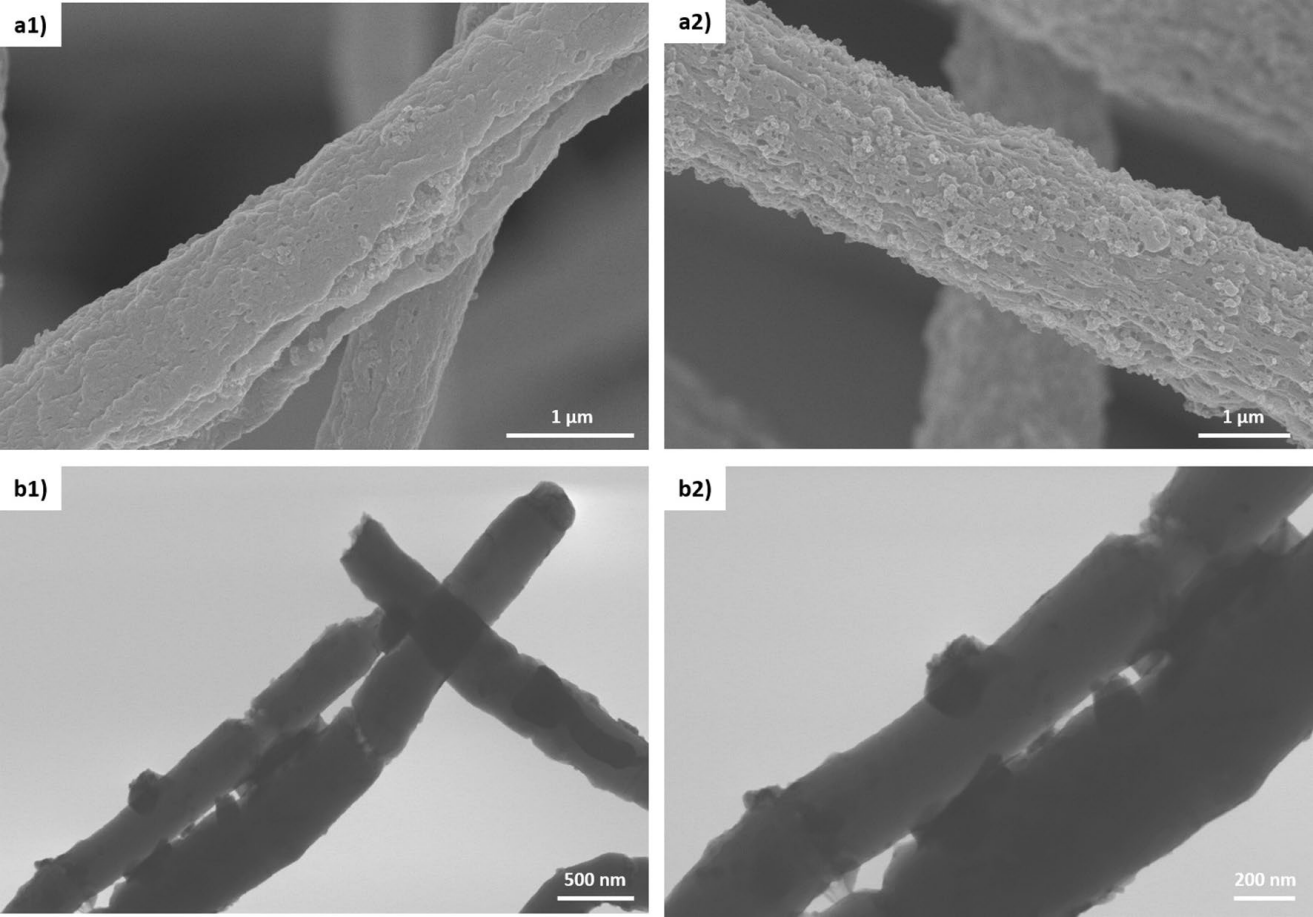
SEM images of CB-loaded nanofibers showing rough surface of fibers when using (**a1**) CB-L and (**a2**) CB-H. STEM images of *as-spun* CB-loaded PAN nanofibrous electrodes with carbon content of ~ 14% at two different magnification: (**b1**) 30,000 and (**b2**) 60,000. CB particles are visible on the surface of the nanofibers.

Since the specific surface area of pure PAN nanofibers (fiber diameter ~ 230 nm) was ~ 33 times higher compared to the commercial carbon felts (~ 10 μm fiber diameter^37^), it could be attributed to its 40-fold smaller fiber diameter. However, the outstanding specific surface area of CB-loaded PAN nanofibers could not only be attributed to their smaller fiber diameter (they had even larger diameter than pure PAN nanofibers; see Fig. 1) but also to the presence of CB in these nanofibers. This larger surface area of CB-loaded fibers could originate from the large surface area of the loaded carbon black itself that had a significant degree of micro-porosity (~ 30% of total surface area)^51^ in combination with superior surface roughness of the fibers (Fig. 3a) which provided additional measured surfaces by the presence of a mesoporous structure^52^.

In addition, as shown in Fig. 3b, the loaded carbon blacks were not only distributed within the fibers, but were also located on the surface of the nanofibers that allowed them to be accessible to the Kr gas during physisorption investigations.

Surprisingly, the *as-spun* fibers loaded with CB-L-2 (with ~ 5 times higher BET of its CB than CB-H, see Table S1, and less mean fiber diameter, see Fig. 1) resulted in a surface area of 66.4 m^2^ g^−1^ which was smaller than that of fibers loaded with CB-H. This result indicated that the amount of loaded CB within fibers and its effect on the fiber morphology had a stronger impact on the BET values when less CB with higher BET values was loaded. See Table S3 for the detailed BET values.

Interestingly, the BET surface area reached a maximum value at 1,000 °C, indicating that carbonization was beneficial for obtaining high surface areas, particularly when CB-L was incorporated: it showed a remarkable ~ tenfold increase from 35.7 to 356.7 m^2^ g^−1^ after carbonization. In general, carbonization led to the formation of a microporous structure^53^ due to the selective removal of PAA and the conversion of PAN to carbon that resulted in higher specific surface areas (Fig. S2 shows the pore size distribution of the carbonized samples from N_2_ physisorption measurements). This observation was reported in earlier works^54,55^ when binary blends of PAN and a sacrificial polymer were utilized for electrospinning.

However, the samples loaded with CB-H-1 showed an increase of surface area from 83.8 m^2^ g^−1^ for *as-spun* fibers to 102.8 m^2^ g^−1^ for carbonized ones, which was only ~ 1.2 times higher. When CB with high content and low BET was loaded (CB-H), they were completely incorporated into the fiber structure. Consequently, no further enhancement of the surface area could be obtained after carbonization (by converting PAN to CNFs). Contrary, the CBs of group L with ~ 14% weight ratio of fabricated fibers and were distributed as isolated aggregates within fibers (Fig. 3b), then connected by CNFs upon carbonization. Therefore, when CB with low content but high BET was chosen (CB-L), the carbonization had a drastic effect on the improvement of BET due to the presence of additional porous and small carbon fibers (Fig. S3).

To summarize, our results indicated that the effect of CB on increasing the surface area of electrospun fibers was significant: when CB was loaded into fibers (e.g. CB-L, CB-H), the BET was higher than PAN-based samples (CB-free). However, this enhancement was affected by the type and amount of the loaded CB in cooperation with the interactions between CB and PAN/PAA.

### Raman data and analysis

**Figure 4:**
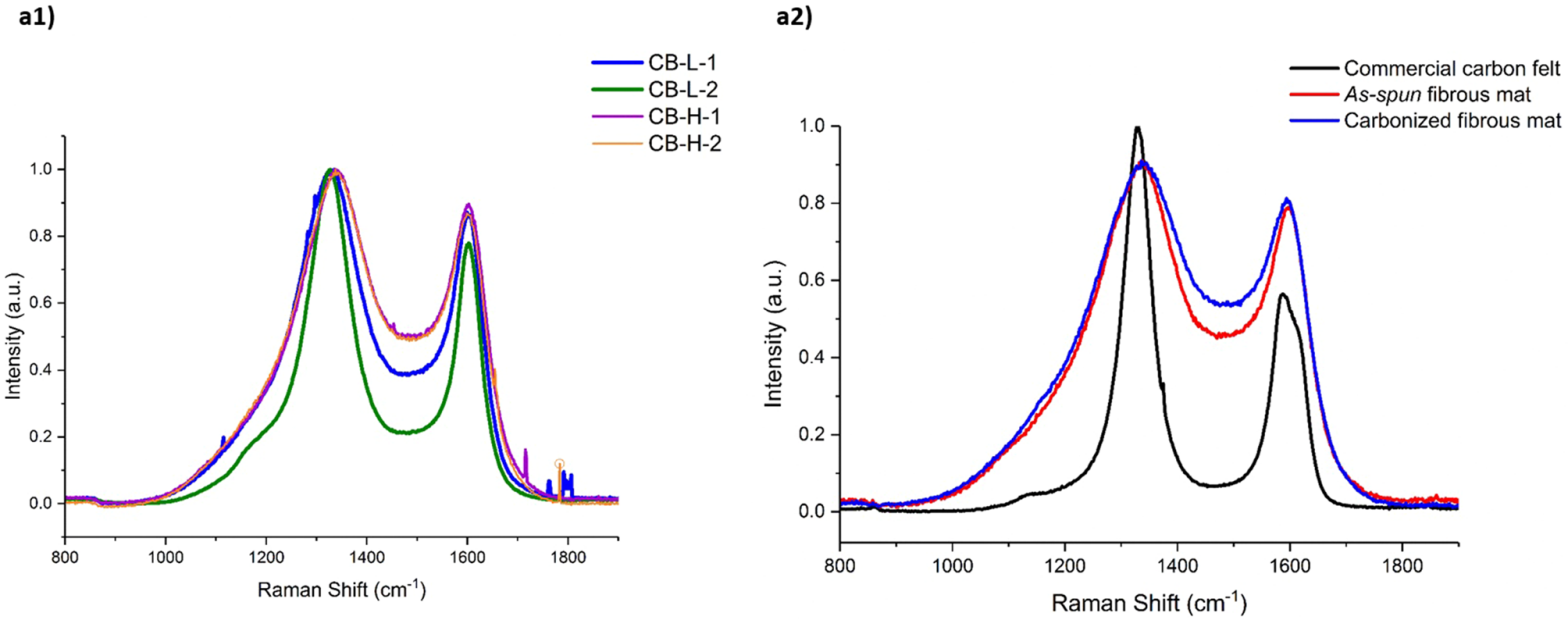
(**a1**) The Raman spectra of various CB: group L and group H, (**a2**) The Raman spectra of commercial carbon felt, *as-spun* nanofibrous mats and carbonized nanofibrous mats.

Figure 4 shows the first-order Raman spectra with the typical appearance of highly disordered carbon nanomaterials with two distinct bands centered at ~ 1,329 to 1,344 cm^−1^ and ~ 1602 to 1613 cm^−1^, corresponding to the D-band (i.e. disordered/defect portion) and the G-band (i.e. ordered structure), respectively. To obtain accurate spectroscopic parameters, a curve-fitting procedure with mixed Gaussian/Lorentzian peaks was conducted for the shown Raman spectra, data displayed in Fig. S4 and Fig. S5. The spectroscopic parameters obtained by curve-fitting the peaks are presented in Table S4.

The lower I_D_/(I_D_ + I_G_) ratio is commonly associated with an ordering and the decrease in the concentration of defects^56^. As shown in Fig. 4a1 and Table S4, carbon blacks of group L presented slightly smaller ratios of the integrated intensity of the D- and G-bands (i.e. ~ 0.73) than CB of group H (i.e. ~ 0.80). This suggested that CB-H had slightly more disordered non-graphitizable structures (more defect intensity) than CB-L. The Raman bands of the CB-H were broader than those of CB-L as a result of the presence of a less ordered structure due to the manufacturing properties.

Interestingly, we observed a noticeable difference between the Raman spectra of the electrospun samples used in this study and the commercial felt (Fig. 4a2, Fig. S5). Remarkably, D2 band (attributed to the E2g stretching mode symmetry of the disordered graphitic lattice located on the surface graphene layers^57^) was not observed in the electrospun samples. Instead, D3 band (originated from the amorphous carbon fraction^57^) and D4 band (related to the (C–C) and (C = C) stretching vibrations or the A1g symmetry mode of the disordered graphitic lattice^57^) were observed for the electrospun fibers. Furthermore, we observed upshifts (~ 12 to 15 cm^−1^ for D band and ~ 22 cm^−1^ for G band) and broadening in Raman bands of electrospun mats compared to the commercial felt that could be attributed to a molecular hydrostatic pressure exerted by the surrounding media^58^. The polymer chains around loaded CBs and various internal and external forces during electrospinning process most likely exerted these compressive forces resulting in nanofibers with a more ordered structure.

The electrospun nanofibrous electrodes (I_D_/(I_D_ + I_G_) ~ 0.68 for carbonized samples) had a lower I_D_/(I_D_ + I_G_) ratio than the commercial carbon felt (I_D_/(I_D_ + I_G_) ~ 0.71) which indicated improved ordered structure (Fig. 4a2). In the commercial felt, the G band exhibited a shoulder at ~ 1613 cm^−1^ typical of defective graphite-like materials. The reason for this different behaviour could be explained by the major structural difference between the electrospun and commercial felts in terms of fiber morphology and components.

### Electrochemical activity for positive half-cell of VRFBs

**Figure 5:**
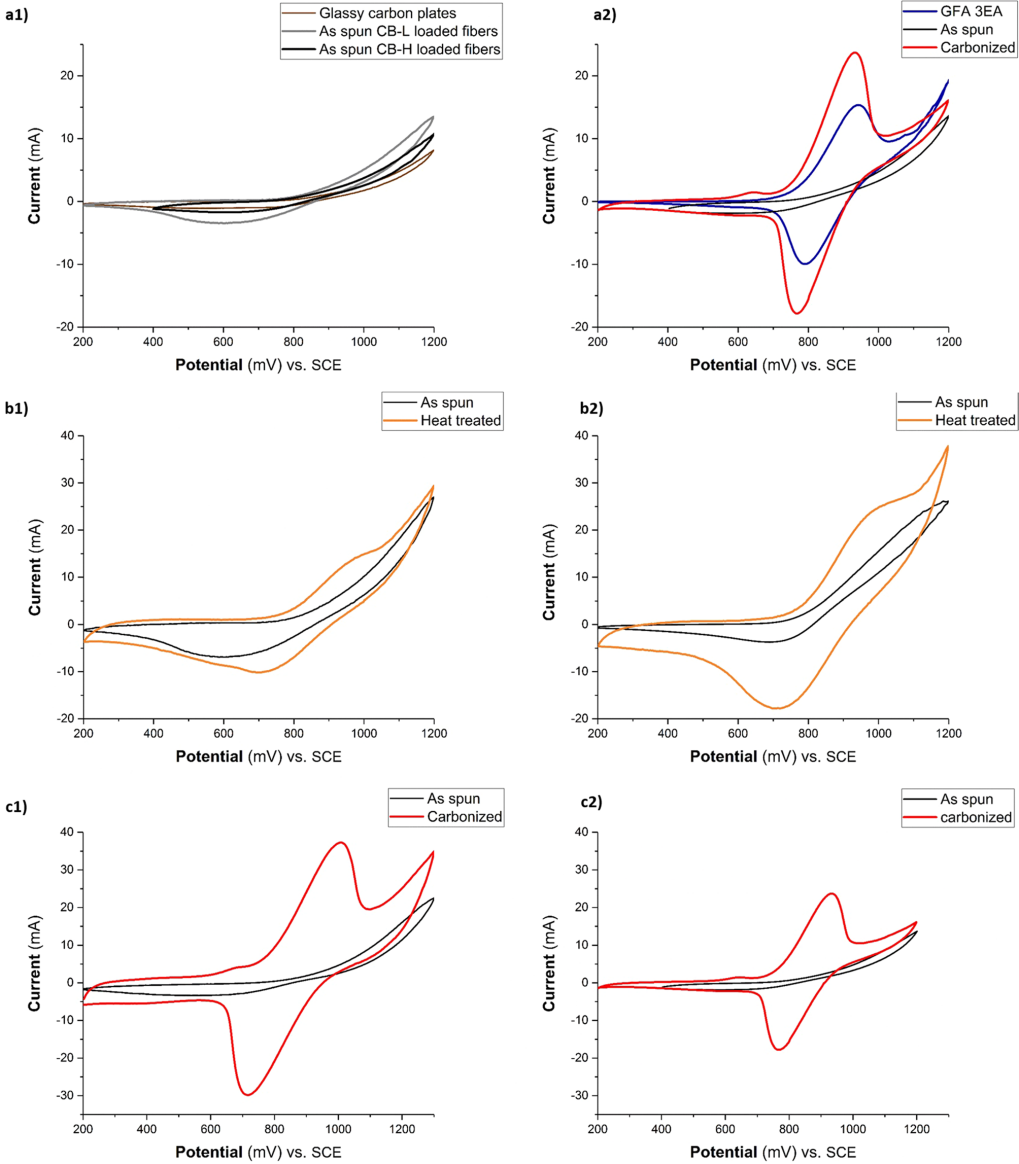
Cyclic voltammograms of (**a1**) the used glassy carbons without employing any sample as reference vs. as-spun fibres sample loaded with two groups of CBs, (**a2**) commercial felt in comparison with as-spun CB-loaded and carbonized CB-loaded electrospun fibrous felts. CB-H was used to fabricate fibers. (**b1**), (**b2**) heat treatment was done at 300 °C in air for 24 h for fibers loaded with CB-L-1 and CB-H-1. (**c1**), (**c2**) carbonization was done at 1,000 °C in inert atmosphere for 1 h for fibers loaded with CB-L-2 and CB-H-2. All cyclic voltammograms were measured for the positive electrode reaction (V^4+^/V^5+^) at a scan rate of 2 mV s^−1^.

The electrochemical activity of various samples towards the V^4+/^V^5+^ redox reaction (i.e., positive-half cell reaction for VRFBs) was investigated by recording their respective cyclic voltammograms in a three-electrode cell. The *as-spun* CB-loaded fibrous electrode (vs. blank glassy carbons used as reference) showed no activity for the V^4+/^V^5+^ redox reaction, as revealed from its recorded featureless CV (Fig. 5a1). The poor activity of the untreated nanofibers despite the presence of CB could be attributed to the large inner-aggregate gap between loaded CB particles within fibers (see Fig. 3) resulting in reduced percolation and thereby electron conductivity. Figure 5a2 shows the cyclic voltammogram (CV) comparison between the commercial carbon felt (CF) and electrospun fibers at a scan rate of 2 mV s^−1^. An improvement in the electrochemical activity towards the V^4+/^V^5+^ reaction was observed for the carbonized electrospun fibers compared to the commercial carbon felt as demonstrated by a significant increase of the V^4+/^V^5+^ oxidation current density with smaller peak separation. The carbonized electrospun sample showed oxidation and reduction peaks at 930 mV and 769 mV versus SCE, respectively. However, these peaks occurred at 940 mV and 791 mV for the commercial carbon felt. For convenient comparison, the changes of current and potential interval of anodic peak and cathodic peak are shown in Table S5. Moreover, the use of electrospun felts exhibited a noticeable improvement with clear distinguished redox peaks in the electrochemical activity toward the V^2+^/V^3+^ reaction (Fig. S6 and Table S6).

This improved electrochemical behavior of carbonized electrospun electrodes compared to the commercial carbon felt could be caused by improved electron transfer and sufficient vanadium redox active sites owing to the enhanced surface area of electrospun mats made of nanofibers that provided abundant active sites for redox reaction (see BET surface area results, Table S3).

Nevertheless, there was no electrochemical activity when these *as-spun* CB-loaded fibers were employed (Fig. 5a). On the other hand, the positive effect of CB on the conductivity had been already observed by other researchers^59^ but polymeric matrices typically require high contents of CB to achieve sufficient electrical conductivity^60^ that is essential for improving electrochemical performance. On the contrary, these high CB concentrations when used for ES solution could produce a highly viscous solution that would terminate the fiber fabrication during in-situ electrospinning process. To mitigate these problematic issues, herein, a post-treatment was suggested to yield a compromise between appropriate polymer-CB matrix processing and its performance for VRFBs application.

Therefore, based on TGA results, the *as-spun* CB-loaded PAN nanofibers were heat treated at 300 °C in open air for 24 h as a mild thermal treatment upon fiber fabrication. This post-treatment on *as-spun* samples at relatively low temperature significantly improved the electrochemical activity towards the positive half-cell reaction of VRFBs (Fig. 5b).

Figure 5b presents the respective CV cycles of CB-loaded nanofibrous electrodes with and without mild heat treatment for two groups of used CB (CB-L and CB-H) at 300 °C in air for 24 h. As obviously seen in this figure, the suggested post-heat treatment improved the electrochemical activity of both electrodes, as demonstrated by the significant negative shift of the oxidation onset potential together with enhancing the reversibility of the V^4+^/V^5+^ redox reaction, see Table S5.

For example, the untreated *as-spun* fibers loaded with CB-H showed almost irreversible behaviour for V^4+/^V^5+^ reaction as easily indicated from the obtained higher oxidation current with very low reduction current (no reduction peak was observed; Fig. 5b2). However, its mild heat treatment, as described above, resulted in a significant increase of its reversibility for V^4+/^V^5+^ redox reactions, as revealed by the appearance of a strong reduction peak. Besides, the peak separation (ΔE) decreased from ~ 500 mV for untreated to ~ 300 mV for heat treated and the anodic to cathodic current ratio (I_pa_/I_pc_) decreased from ~ 7.21 for untreated to ~ 1.45 for heat treated electrode. Moreover, the effect of the CB parameters on ΔE (i.e. ~ 300 mV) was not significantly different for the cyclic voltammograms obtained for the positive electrolyte. This enhanced activity might be the result of favourable interactions between the individual fibrous layers when this mild thermal treatment was applied. Interestingly, the fibers with CB-H exhibited a higher oxidation peak current and reduction peak current (Table S5). This phenomenon could be explained as fibers loaded with CB-H had higher numbers of CB that were well-integrated into fibers (also available on the fiber surface as shown in Fig. 3) with high BET surface area (Table S3) and they could provide a better access for the V^4+/^V^5+^ redox reaction. In addition, since both loaded CBs had diverse morphological properties (Table S1), they made fibers with different morphological characteristics in terms of fiber surface porosity (visible in the Fig. 3) and bulk porosity (due to the difference in fiber diameter as indicated in Fig. 1). Therefore, these different porosities could shift the peak potentials owing to the diffusion limitations in the pores^23,61^.

On the other hand, the above-mentioned heat treatment at 300 °C (as post treatment upon fiber fabrication) drastically improved the thermal properties of the *as-spun* CB-loaded fibrous mat for both types of CBs (Fig. S7). The slight mass loss started at around 500 °C to give a yield of ~ 60% at 1,000 °C which suggested that this heat treatment at 300 °C modified the activity of the free radicals involved in the complex chemical reactions.

Figure 5c displays the recorded CV cycle of CB-loaded nanofibrous electrodes with various types of CB after carbonization at 1,000 °C in inert atmosphere for 1 h. As shown in the Fig. 5c, the oxidation peak current (*I*_pa_) of the redox couples increased when CB-L was loaded: 37.3 mA vs. 23.7 mA for the electrospun fibers loaded with CB-H. As clearly seen, both carbonized CB-loaded fibrous electrodes presented higher electrochemical activity compared to the commercial carbon felt. Interestingly, despite the high loading content of CB-H within fibers (i.e. ~ 3.6 times more than CB-L), the carbonized samples exhibited decreased electrochemical performance (see Table S5) compared to the fibers loaded with CB-L. The fibers loaded with CB-L had better electrochemical activity toward the V^4+/^V^5+^ redox reaction, since it had higher oxidation/reduction peak current (i.e. I_pa_ = 37.3 mA, I_pc_ = 30 mA) accompanied by a ratio of *I*_pa_/*I*_pc_ closer to 1 (i.e. ~ 1.2). This indicated that the carbonization step at 1,000 °C along with the use of CB-L in fibers was the most promising strategy to obtain better electrochemical performance. This observation could be attributed to the extremely high surface area of carbonized sample when CB-L was utilized (i.e. 356.7 m^2^ g^−1^).

However, the same fibers made of CB-L were not as electrochemically active as fibers with CB-H when heat treated at 300 °C. This observation could be explained by the low content of CB-L present in the fibers structure (Fig. 3b) in which despite its intrinsic high surface area (~ 1,400 m^2^ g^−1^), this CB could not exceptionally increase the surface area of fibers (~ 35.7 m^2^ g^−1^ for CB-L-1 and ~ 66.4 m^2^ g^−1^ for CB-L-2) due to its low loading within fibers (~ 14%). Therefore, CB-L was unable to significantly improve the electrochemical performance unless their positive role was amplified by converting PAN into the carbon fibers (which acted as bridge to connect the gaps between low loaded CB-L within fibers) employing a carbonization step at 1,000 °C. Consequently, the role of intrinsic BET of CB over its ratio in the polymer-CB matrix was noticeable when carbonization at 1,000 °C was applied upon fiber fabrication and this trend was opposite when mild heat-treatment at 300 °C was chosen.

Moreover, CB-H-loaded fibers with mild heat treatment exhibited almost the same high peak current of carbonized CB-H-loaded samples (~ 24 mA and ~ 18 mA for the anodic and cathodic processes, respectively), which suggested to utilize this group of CB-loaded fibrous electrodes with a mild heat treatment instead of carbonization at high temperature.

Furthermore, a comparison of the electrochemical activity of the carbon black inks without loading them into nanofibers is presented in Fig. S8: cyclic voltamograms of the CB-inks exhibited an oxidation peak at 925 mV only for CB-L. The higher electrochemical activity of CB-L, where the same amount as CB-H was used, could be due to their specific properties: carbon blacks with higher surface area (CB-L) could provide more particles per unit area (more accessibility) and, therefore, reduce the average gap width between their aggregates. However, the usage of 50% weight ratio of CB-L in the polymer-CB matrix was not feasible when electrospinning was the selected manufacturing process to make these nanofibrous electrodes.

The key determinant of the electrochemical performance was not the electrical conductivity but rather the number of active sites^62^, which was governed by the morphology and the number of defects in the electrode. Since all electrospun CB-loaded fibrous electrodes were prepared and tested under the same technical conditions, the electrochemical performance of the electrolyte could be related to the nanofiber morphology, different structure of the loaded CB and their interactions when various post-treatment strategies were applied. These results suggested that the crucial factor for the electrochemical performance of CB-loaded electrospun fibers was not only a higher number of active sites provided by the nanofibrous morphology of the electrode and presence of CB, but it could be attributed to the fibers’ morphological changes induced by these loaded-CBs and their interactions in CB-polymeric matrix. We conclude that both types of the CB-L could contribute a positive effect on the electrochemical activity of the electrolyte for V^4+/^V^5+^ and V^2+/^V^3+^ redox reactions as long as some heat treatment is employed.

### Full-cell cycling test

**Figure 6:**
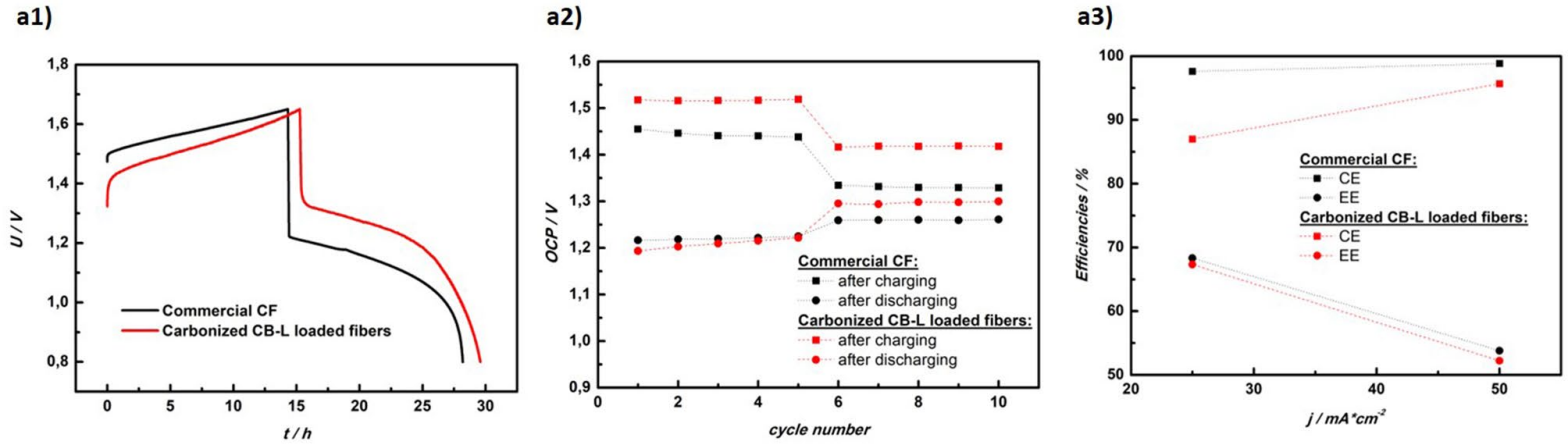
Results of full-cell cycling test. (**a1**) typical charge and discharge voltage curves at 25 mA cm^−2^, (**a2**) open circuit potentials (OCP) obtained after each charging and discharging step, (**a3**) mean efficiencies (Coulombic efficiency as CE, energy efficiency as EE) at 25 and 50 mA cm^−2^.

Results of the full-cell cycling test are shown in Fig. 6. At low current densities of 25 mA cm^−2^, higher capacities could be achieved when using the carbonized electrospun CB-L loaded fibrous electrode compared to the reference material (commercial CF), as indicated by the increased duration of charging and discharging process (Fig. 6a1). This was further underlined by the higher obtained open circuit potentials (OCP) after charging during the first five cycles (Fig. 6a2), meaning that higher states of charge (SOC) were accessible with the electrospun electrode. This outcome was in good agreement with the results of CV and BET measurements (see Fig. 5, Table S5, Table S6 and Fig. S6for CV results and Table S3 for BET values). Based on these findings, we conclude that the enlarged surface area of the electrospun electrode had an enhancing effect on the kinetics of the V^2+^/V^3+^ redox reaction, effectively lowering kinetic overvoltages in the negative half-cell. On the other hand, from Fig. 6a3, it was obvious that higher Coulombic efficiency (CE) and energy efficiency (EE) were obtained when using only commercial CF. The significantly lower Coulombic efficiency for the electrospun material at 25 mA cm^−2^ might, to a certain extent, be explained by the prolonged cycling time, which was found to mitigate vanadium crossover in diffusion-dominated membranes^63^. Since the energy efficiency was still comparable to that of the commercial felt electrode, this meant that the voltage efficiency (VE) was higher for the electrospun material (based on EE = CE × VE). This observation might be due to the catalytic enhancement introduced by utilization of the electrospun CB-L loaded fibrous electrode. However, the lower CE measured in CB-L loaded fibers indicated that side reactions in them were more pronounced compared to the commercial felt electrode. We suspect that an increased mass transport limitation (towards the vanadium reaction) due to the smaller inter-fibre distances and thus smaller pore space could be the reason for this behaviour (Fig. S2 shows microporous structure of the fibrous electrodes particularly when fibers were loaded with CB-L). Since in galvanostatic operation the electrodes had to provide a constant current, limited supply of active species would have a negative effect on the balance between desired vanadium redox reaction and parasitic reactions (like the hydrogen evolution reaction). The subject of future studies will therefore be to circumvent this issue by using a different cell geometry (i.e. flow-by instead of flow-through) and optimizing the inter-fibre distance during electrospinning process.

## Conclusions

Electrospun nanofibrous webs with tuned structure were successfully fabricated by in-situ incorporating of low (~ 14%) and high (~ 50%) contents of carbon black powders with different morphological and physical properties into blends of the polymeric matrix. Addition of CB made a rough surface and increased the fiber diameter from ~ 230 nm (for pristine PAN fibers) to ~ 400 nm (CB-L) and ~ 650 nm (CB-H) for CB-loaded fibers. These interconnected fibrous networks exhibited a good electrochemical response towards both vanadium reactions with an additional heat treatment (300 °C, 24 h, air) or carbonization (1,000 °C, 1 h, N_2_) step following fiber fabrication. Enhanced electrochemical activity of the *as-spun* fibers with mild heat treatment at 300 °C was more promising when CB-H was loaded into PAN-based fibers due to their 50% content as well as fiber diameter and porous morphology. In addition, these heat treated CB-loaded fibers preserve their defect-free fiber morphology providing high surface area and a highly flexible and free-standing 3D fibrous composite electrode. However, the carbonized samples loaded with CB-L showed an excellent electrochemical activity compared to the commercial felt (oxidation and reduction peak current enhanced by factors of 2.4 and 3 respectively) and CB-H loaded fibers.

The incorporation of CB into PAN/PAA enhanced the thermal stability of the fibers to 310 °C (loading CB-L) and 325 °C (loading CB-H) compared to the CB-free samples which was 295 °C and this effect was more pronounced when CB-H was employed due to its intrinsic high thermal stability. Interestingly, heat treated fibers at 300 °C presented extremely high thermal stability (up to 500 °C). Raman spectroscopy studies showed that the CB-loaded fibers, particularly when CB-L was used, were more ordered and had less defects compared to the available commercial carbon felt.

This fibrous construct utilized as electrode leads to an improved electrochemical activity towards V^4+/^V^5+^ and V^2+/^V^3+^ redox reactions as a higher reversibility, a lower anodic potential peak value and a higher current for the reaction are provided. Furthermore, the ful-cell cycling test at low current density of 25 mA cm^−2^ showed promising results in terms of achievable capacity and voltage efficiency towards the kinetics of the V^2+/^V^3+^ redox reaction when a CB-L loaded electrospun fibrous electrode was employed. This study may pave the way for developing dedicated engineering strategies for fibrous-based materials for positive electrodes in VRFBs.

## Supplementary information

Supplementary information
