# Supplementary material for: Fabrication of an efficient vanadium redox flow battery electrode using a free-standing carbon-loaded electrospun nanofibrous composite
Source: Sci Rep. 2020 Jul 7;10:11153. doi: 10.1038/s41598-020-67906-6 (PMC7340777; doi:10.1038/s41598-020-67906-6)
Supplement: Supplementary file 1 — Supplementary information [file 41598_2020_67906_MOESM1_ESM.docx]

Fabrication of an Efficient Vanadium Redox Flow Battery Electrode Using a Free-standing Carbon-loaded Electrospun Nanofibrous Composite

Mahboubeh Maleki*^a^, Gumaa A. El-Nagar ^ab^, Denis Bernsmeier ^c^, Jonathan Schneider ^a^ and Christina Roth *^ad^

*^a^ Institute for Chemistry and Biochemistry, Freie Universität Berlin, Berlin 14195, Germany*

*^b^ Chemistry Department, Faculty of Science, Cairo University, Cairo 12613, Egypt*

*^c^ Department of Chemistry, Chemical Engineering Division, Technical University of Berlin, Berlin 10623, Germany*

*^d^* *Energy Materials Engineering, Faculty of Engineering, University of Bayreuth, 95447 Bayreuth, Germany*

**Corresponding authors’ email:* [*maleki.m83@gmail.com*](mailto:maleki.m83@gmail.com) *; christina.roth@uni-bayreuth.de*

**Table S1.** Morphological properties of used carbon blacks in this study

|  | Carbon Black | CB label | Particle Diameter (nm) | Specific Surface Area (m² g^-1^) |
| --- | --- | --- | --- | --- |
| Group L | XCmax | CB-L-1 | - | 1350 |
|  | Ketjenblack EC600JD | CB-L-2 | 30-100 | 1400 |
| Group H | XC72 | CB-H-1 | 30-60 | 230-250 |
|  | Printex L6 | CB-H-2 | 18 | 270 |

**Table S2.** Electrospinning solutions preparations for fabrication of CB-loaded fibrous composite

|  | Amount of PAN (g) | Amount of PAA (g) | Amount of CB (g) | DMF (mL) | Total concentration (wt%) | Weight Ratio (PAN:PAA:CB) |
| --- | --- | --- | --- | --- | --- | --- |
| CB-free fibers | 0.29 | - | - | 3 | 9.3 | 100%:0:0 |
| CB-L loaded fibers | 0.27 | 0.03 | 0.05 | 3 | 11 | 77%:9%:14% |
| CB-H loaded fibers | 0.27 | 0.03 | 0.3 | 3 | 17.5 | 45%:5%:50% |

**Table S3.** BET results of physisorption measurements to evaluate surface area of fabricated fibrous electrodes

| Samples | Type of sample | Specific Surface Area (m² g^-1^) |
| --- | --- | --- |
| SGL GFA 3EA | Commercial carbon felt | 0.6 |
| CB-free fibers | *As-spun* | 15.5 |
| CB-free fibers | Carbonized | 19.8 |
| CB-L-1 loaded fibers | *As-spun* | 35.7 |
| CB-L-1 loaded fibers | Carbonized | 356.7 |
| CB-L-2 loaded fibers | *As-spun* | 66.4 |
| CB-L-2 loaded fibers | Carbonized | 275.1 |
| CB-H-1 loaded fibers | *As-spun* | 83.8 |
| CB-H-1 loaded fibers | Carbonized | 102.8 |
| CB-H-2 loaded fibers | *As-spun* | 61.7 |
| CB-H-2 loaded fibers | Carbonized | 99.1 |


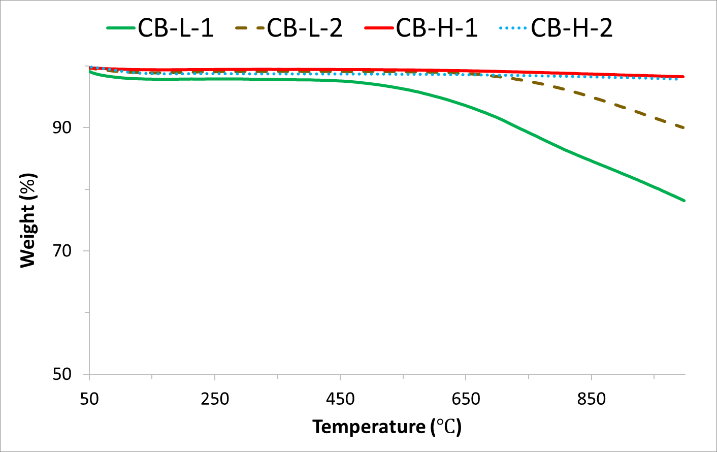


**Fig. S1.** TGA profile of received CB: L-1, L-2, H-1 and H-2





**Fig. S2.** The pore size distribution from N_2_ physisorption of the carbonized CB-loaded nanofibers showing microporous structure of the fibers particularly when loading CB-L.


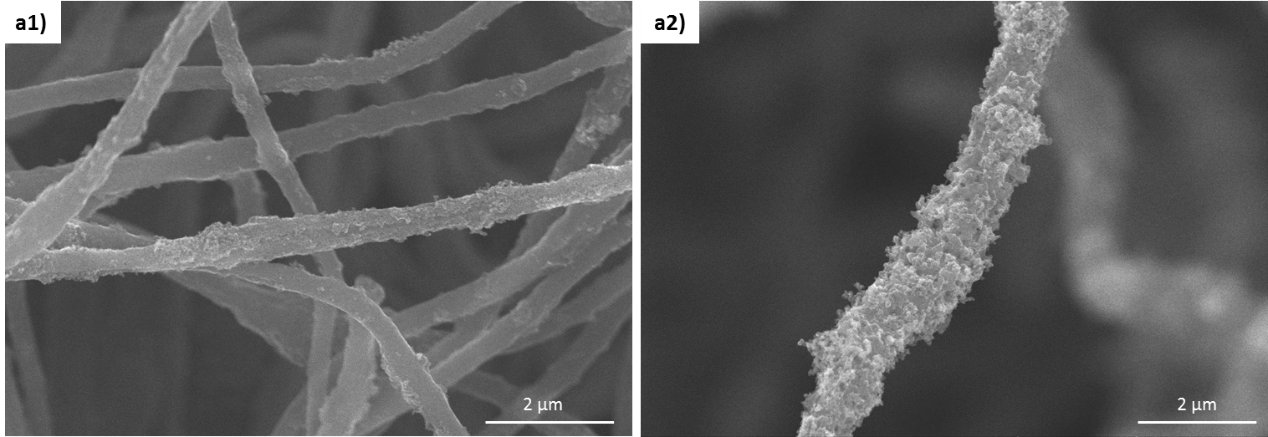


**Fig. S3.** SEM images of carbonized CB-loaded nanofibers showing rough and porous surface of fibers when using a1) CB-L, fiber diameter 239 ± 105 nm and a2) CB-H, fiber diameter 593 ± 173 nm


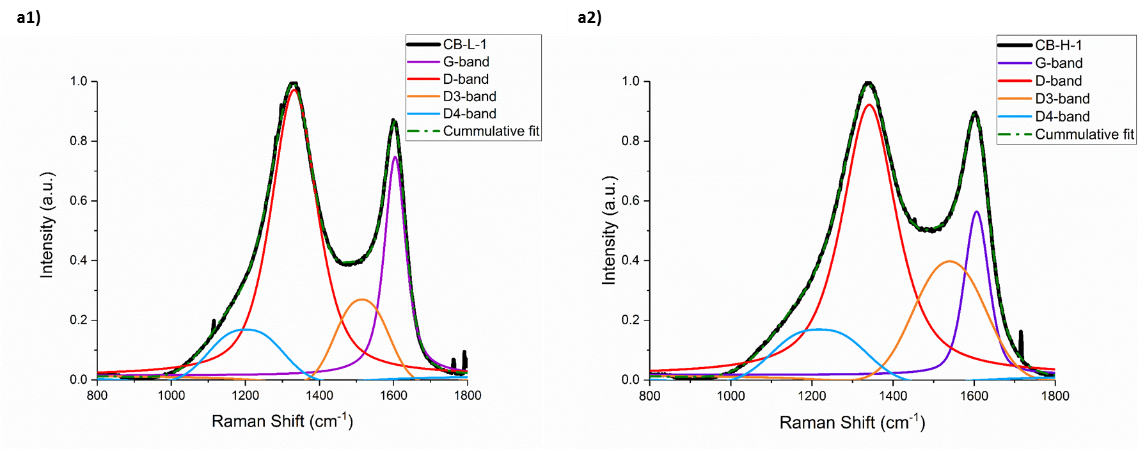


**Fig. S4.** Cumulative fits of the Raman spectra of two different carbon blacks: a1) CB-L-1, a2) CB-H-1


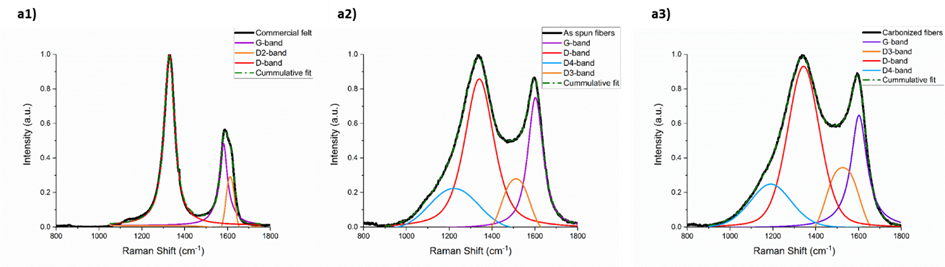


**Fig. S5.** Cumulative fits of the Raman spectra for: a1) commercial carbon felt (SGL GFD 3EA), a2) *as-spun* nanofibrous mat, a3) carbonized nanofibrous mat

**Table S4.** Raman spectroscopic parameters of the different types of carbon blacks and commercial carbon felt vs. electrospun samples

| Samples |  | Position (cm^-1^) | Intensity (area) | FWHM | I_D_/ (I_D_+I_G_) |
| --- | --- | --- | --- | --- | --- |
| CB-L-1 | **D** | **1332** | **192** | 147 | 0.73 |
|  | D3 | 1515 | 24 | 152 |  |
|  | D4 | 1201 | 15 | 203 |  |
|  | **G** | **1605** | **71** | 69 |  |
| CB-H-1 | **D** | **1341** | **206** | 160 | 0.80 |
|  | D3 | 1540 | 62 | 196 |  |
|  | D4 | 1218 | 12 | 230 |  |
|  | **G** | **1606** | **50** | 71 |  |
| GFA 3EA | **D** | **1329** | **90** | 60 | 0.71 |
|  | D2 | 1613 | 9 | 43 |  |
|  | **G** | **1581** | **37** | 46 |  |
| *As-spun* | **D** | **1341** | **191** | 163 | 0.67 |
|  | D3 | 1512 | 26 | 136 |  |
|  | D4 | 1223 | 60 | 263 |  |
|  | **G** | **1603** | **96** | 85 |  |
| carbonized | **D** | **1344** | **202** | 178 | 0.68 |
|  | D3 | 1526 | 28 | 158 |  |
|  | D4 | 1190 | 69 | 229 |  |
|  | **G** | **1602** | **94** | 91 |  |

**Tabel S5.** Measured values from cyclic voltammograms of the *as-spun* heat treated electrospun fibrous felts for the positive electrode reaction (V^4+^/V^5+^)

|  | I _pa_ (mA) | I _pc_ (mA) | E/V_pa_ (mV) | E/V_pc_ (mV) | −I_pa_/I_pc_ | Δ*E* (mV) |
| --- | --- | --- | --- | --- | --- | --- |
| Commercial carbon felt vs. carbonized electrospun sample | | | | | | |
| Commercial | 15.38 | -9.96 | 940 | 791 | 1.54 | 149 |
| carbonized | 23.7 | -17.85 | 930 | 769 | 1.33 | 161 |
| Heat treatment was done at 300℃ in air for 24 hours. | | | | | | |
| PAN PAA CB-L-1 | 14.89 | -10.26 | 999 | 699 | 1.45 | 300 |
| PAN PAA CB-H-1 | 24.36 | -17.75 | 990 | 712 | 1.37 | 278 |
| Carbonization was done at 1000℃ in inert atmosphere for 1 hour. | | | | | | |
|  | I _pa_ (mA) | I _pc_ (mA) | E/V _p_ (mV) | E/V_a_ Min (mV) | −I_pa_/I_pc_ | Δ*E* (mV) |
| PAN PAA CB-L-2 | 37.26 | -29.99 | 1008 | 716 | 1.24 | 292 |
| PAN PAA CB-H-1 | 23.7 | -17.85 | 930 | 769 | 1.33 | 161 |

**Tabel S6.** Measured values from cyclic voltammograms of the electrospun fibrous felts (PAN PAA CB-L-2) for the negative electrode reaction (V^2+^/V^3+^)

|  | I _pa_ (mA) | I _pc_ (mA) | E/V_pa_ (mV) | E/V_pc_ (mV) | −I_pa_/I_pc_ | Δ*E* (mV) |
| --- | --- | --- | --- | --- | --- | --- |
| Heat treated at 300℃ | 8.50 | -11.09 | -389 | -641 | 0.77 | 252 |
| Carbonized at 1000℃ | 16.57 | -23.71 | -435 | -609 | 0.70 | 174 |


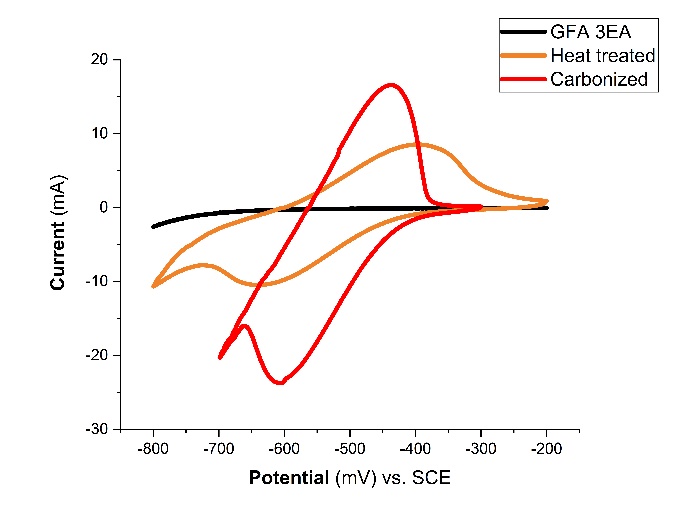


**Fig. S6.** Cyclic voltammograms of the commercial carbon felt and electrospun CB-L loaded electrodes for the negative electrode reaction (V^2+^/V^3+^). All samples utilized as working electrode were ~8 mg. The cell was filled with 0.2 mol L^-1^ vanadium (51% V^3+^ and 49% V^4+^) in 2 mol L^-1^ H_2_SO_4_ electrolyte. The CV measurements were performed over a potential range from -800 mV to -200 mV at a scan rate of 2 mV s^−1^.


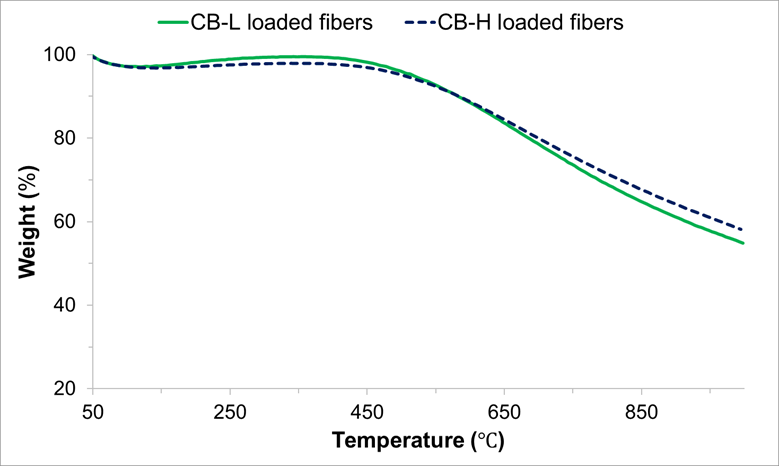


**Fig. S7.** TGA curves of *as-spun* heat-treated nanofibrous electrodes with CB: PAA:CB-L and PAN:PAA:CB-H. Heat treatment was done at 300℃ in air for 24 hours


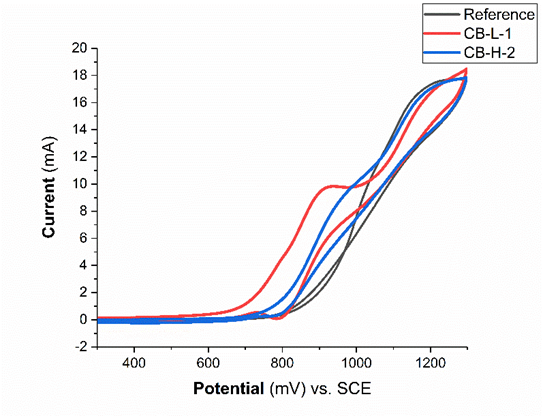


**Fig. S8.** Cyclic voltammograms of the CB-inks compared to the reference (blank carbon paper) for the positive electrode reaction (V^4+^/V^5+^) measured at a scan rate of 2 mVs^-1^. CBs were from different groups (L and H). To make CB-ink: 7 mg of CB was dissolved in isopropanol (1) : water (1) (v:v) mixture and then 100 µL Nafion was added and then sonicated for 1 h. Then 40 µL of this solution was placed on a carbon paper for further analysis
